# Multimodal Digital Phenotyping Study in Patients With Major Depressive Episodes and Healthy Controls (Mobile Monitoring of Mood): Observational Longitudinal Study

**DOI:** 10.2196/63622

**Published:** 2025-02-21

**Authors:** Talayeh Aledavood, Nguyen Luong, Ilya Baryshnikov, Richard Darst, Roope Heikkilä, Joel Holmén, Arsi Ikäheimonen, Annasofia Martikkala, Kirsi Riihimäki, Outi Saleva, Ana Maria Triana, Erkki Isometsä

**Affiliations:** 1 Department of Computer Science Aalto University Espoo Finland; 2 Department of Psychiatry University of Helsinki Helsinki Finland; 3 Helsinki and Uusimaa Hospital District Helsinki Finland; 4 School of Science Aalto University Espoo Finland; 5 City of Helsinki Mental Health Services Helsinki Finland; 6 University of Turku and Turku University Central Hospital Turku Finland; 7 Finnish Institute for Health and Welfare Helsinki Finland

**Keywords:** digital health, mental disorders, depression, digital phenotyping, smartphones, mobile devices, multisensor, mobile phone

## Abstract

**Background:**

Mood disorders are among the most common mental health conditions worldwide. Wearables and consumer-grade personal digital devices create digital traces that can be collected, processed, and analyzed, offering a unique opportunity to quantify and monitor individuals with mental disorders in their natural living environments.

**Objective:**

This study comprised (1) 3 subcohorts of patients with a major depressive episode, either with major depressive disorder, bipolar disorder, or concurrent borderline personality disorder, and (2) a healthy control group. We investigated whether differences in behavioral patterns could be observed at the group level, that is, patients versus healthy controls. We studied the volume and temporal patterns of smartphone screen and app use, communication, sleep, mobility, and physical activity. We investigated whether patients or controls exhibited more homogenous temporal patterns of activity when compared with other individuals in the same group. We examined which variables were associated with the severity of depression.

**Methods:**

In total, 188 participants were recruited to complete a 2-phase study. In the first 2 weeks, data from bed sensors, actigraphy, smartphones, and 5 sets of daily questions were collected. In the second phase, which lasted up to 1 year, only passive smartphone data and biweekly 9-item Patient Health Questionnaire data were collected. Survival analysis, statistical tests, and linear mixed models were performed.

**Results:**

Survival analysis showed no statistically significant difference in adherence. Most participants did not stay in the study for 1 year. Weekday location variance showed lower values for patients (control: mean –10.04, SD 2.73; patient: mean –11.91, SD 2.50; Mann-Whitney *U* [MWU] test *P*=.004). Normalized entropy of location was lower among patients (control: mean 2.10, SD 1.38; patient: mean 1.57, SD 1.10; MWU test *P*=.05). The temporal communication patterns of controls were more diverse compared to those of patients (MWU test *P*<.001). In contrast, patients exhibited more varied temporal patterns of smartphone use compared to the controls. We found that the duration of incoming calls (β=–0.08, 95% CI –0.12 to –0.04; *P*<.001) and the SD of activity magnitude (β=–2.05, 95% CI –4.18 to –0.20; *P*=.02) over the 14 days before the 9-item Patient Health Questionnaire records were negatively associated with depression severity. Conversely, the duration of outgoing calls showed a positive association with depression severity (β=0.05, 95% CI 0.00-0.09; *P*=.02).

**Conclusions:**

Our work shows the important features for future analyses of behavioral markers of mood disorders. However, among outpatients with mild to moderate depressive disorders, the group-level differences from healthy controls in any single modality remain relatively modest. Therefore, future studies need to combine data from multiple modalities to detect more subtle differences and identify individualized signatures. The high dropout rates for longer study periods remain a challenge and limit the generalizability.

## Introduction

### Background

Mood disorders, including major depressive disorder (MDD) and bipolar disorder (BD), are substantial contributors to the worldwide disease burden and impairment, bearing important implications for public health [1,2]. They also have an increased association with premature mortality [3,4]. Mood disorders are usually recurring and often chronic conditions that typically require long-term treatment [5]. Currently, the diagnostic landscape and assessment of symptom severity for mood disorders are primarily based on clinical evaluations, interviews, and questionnaires, which are predominantly reliant on subjective interpretations and retrospective recollections by patients and only conducted when the patient visits the mental health professionals. This framework is susceptible to memory bias and may not capture the dynamic symptomatology inherent in mood disorders [6,7]. Compounded by the lack of clear biomarkers and a worldwide shortage of adequate mental health resources, the treatment of these conditions has long posed challenges.

In recent years, the ubiquity of digital technologies, particularly wearables and personal digital devices, has facilitated their integration into different fields of medicine. These devices can measure various physiological metrics, such as heart rate, heart rate variability, and respiration rate [8]. Moreover, through interactions with these devices, significant amounts of digital traces are generated, which can be collected, analyzed, and transformed into valuable information regarding their users’ behavior and activities. In the case of psychiatric disorders, particularly mood disorders, leveraging digital traces and the associated information holds the potential to introduce greater objectivity in diagnosing and treating these conditions as well as the possibility for continuous monitoring of patients. The emerging field of digital phenotyping harnesses real-time indicators of human behavior to uncover behavioral markers that objectively quantify, monitor, and assess mental health conditions continuously [9-14].

Several digital phenotyping studies have used data collected from personal digital devices to extract behavioral features. These studies typically use clinically validated scales, such as the 9-item Patient Health Questionnaire (PHQ-9) [15], to measure symptoms of the disorder, using them as a reference point. Most digital phenotyping studies aim to determine which passive behavioral features, gathered as participants interact with their devices, are most indicative of clinical scores. A recent review [16] examined 45 articles and found that most of these studies focus on a single modality. In contrast, a multimodal approach could provide a more accurate understanding of an individual’s life and behaviors. In addition, many of the studies involve nonclinical cohorts or individuals who self-identified as experiencing depression through self-administered questionnaires, rather than individuals formally diagnosed with depression.

### Objectives

In response to significant challenges in digital phenotyping, we designed and conducted the Mobile Monitoring of Mood (MoMo-Mood) study. These challenges include the scarcity of research involving patients with clinically diagnosed mood disorders, the necessity for prolonged observation periods to identify behavioral precursors to recurrent episodes in mood disorders, and the requirement for a multimodal approach to capture behaviors from various data streams. This multimodal digital phenotyping study focused on different types of patients who were receiving treatment for major depressive episodes, including (unipolar) MDD, BD, or borderline personality disorder (BPD), as well as healthy controls. Before MoMo-Mood, we conducted a smaller pilot study (MoMo-Mood Pilot) to test the feasibility of our study design before scaling it to a larger number of participants and patients with less prevalent disorders. In this work, we explore the potential and effectiveness of personal digital devices in capturing clinically significant behavioral indicators from patients with diagnosed conditions over (a maximum of) a period of up to 1 year. The unique combination of patient subcohorts, a wide range of data streams from different devices, and an extended study time allows us to, for the first time, test the feasibility of such a study design at a larger scale and gain insights into what works and what needs to be improved for future studies.

While study adherence has been recognized as a challenge in digital phenotyping studies [17], how different groups of patients comply with long-term passive data collection remains understudied. Similarly, while past research has suggested that patients with serious mental disorders have similar or even higher rates of smartphone use compared to others [18,19], the frequency and quantity of smartphone use in different groups of mood disorders need to be better examined. To this end, we measured adherence based on the time each person has remained in the study (maximum 1 year). Furthermore, we used smartphone screen activity (on, off, lock, and unlock) time stamps as a proxy for general smartphone use and smartphone apps used for specific types of use.

In addition to that, we hypothesized that patients (compared to healthy controls) exhibited differences when comparing activities and behaviors that were relevant for assessing mood disorder symptoms or associated psychosocial disability. Here, we focused on 4 domains: social activity, mobility, physical activity, and sleep. Depression is known to be associated with social isolation [20], which can potentially be reflected in a person’s communication pattern. Fatigue and loss of energy are among the diagnostic criteria for depression [21,22], which can be reflected in mobility and physical activity data. Past research has used passively collected location data from smartphones. It has identified variables such as location variance, location entropy, and time spent at home to be associated with symptoms of depression [23-25]. Finally, both increased and decreased sleep are symptoms of depression [26]. In our study, as a proxy of social activity, we used time stamps for incoming and outgoing calls and SMS text messages through the phone network provider. Location and mobility were measured through the smartphone’s GPS and Wi-Fi data, and the variables shown to exhibit significant differences between patients and healthy controls in other studies were investigated. We measured physical activity in 2 ways: first, through actigraphs, and second, through smartphone accelerometers. Sleep timing and duration were measured by actigraphs and bed sensors.

Moreover, to assess several different behaviors, we focused on their temporal patterns by exploring activity rhythms and how patients and controls allocated their time to a particular activity throughout the week. The timing of different activities may contain important behavioral markers linked to mood disorders. Previous research in studying temporal rhythms of activity inferred from different personal digital devices has shown that people tend to have persistent activity rhythms over time [27-30]. However, these rhythms and their consistency are linked to personal and sociodemographic characteristics [31,32]. A recent study [33] suggests that daily activity rhythms inferred from smartphone GPS data are associated with anxiety levels in patients with MDD and BD. While we know that mood disorders impact daily activity and sleep rhythms, the activity rhythms of patients with mood disorders inferred from passively collected data from their smartphones are not well studied [34]. This work investigates the different activity rhythms inferred from smartphones and evaluates the differences between patients and healthy controls.

We specifically aim to answer the following research questions (RQs):

- RQ1: Does study adherence, that is, how long the participants remain in the study, vary between patient subcohorts and healthy controls?
- RQ2: Do the quantity and temporal patterns of smartphone use, in terms of phone screen and app use, vary between patients and controls?
- RQ3: Do differences exist between patients and healthy controls when comparing social activity, mobility, physical activity, and sleep, as measured by passively collected data?
- RQ4: To what degree do people within the same subcohort show similar temporal rhythms of smartphone use, social activity, and physical activity?
- RQ5: Is the severity of depression symptoms during the follow-up associated with social activity, mobility, physical activity, and sleep?

## Methods

### Setting

We initially conducted a smaller-scale pilot study, the MoMo-Mood Pilot, to test our study design. Once the study was deemed feasible, we moved on to a larger-scale study, namely, MoMo-Mood. Other than the number of participants, the main difference between the 2 studies was that for the pilot study, participants were either patients with MDD or healthy controls, as opposed to the main study, where 2 additional patient subcohorts were recruited. The recruitment of all the cohorts for the main study was performed independently from the pilot study. In addition, the pilot study had a shorter maximum data collection period from patients (6 months) than the MoMo-Mood study (1 year).

Both studies were conducted in Finland. Patients who had already been diagnosed using structured clinical interviews were recruited on a voluntary basis from the mood disorder outpatient treatment facilities at the Helsinki University Hospital Mood Disorder Division, Turku University Central Hospital Department of Psychiatry, and City of Espoo Mental Health Services. The Mini International Neuropsychiatric Interview [35] was used for the diagnosis of MDD and BD, and the Structured Clinical Interview-II [36] was used for BPD. The patients with any psychotic features, concurrent substance use disorder, or imminent risk of suicide were excluded.

The studies had an active phase that lasted 2 weeks, during which we collected data from actigraphs and bed sensors loaned to study participants. Different types of data were passively collected (passive data) from smartphone sensors through a smartphone app. Passive data are a byproduct of the participants’ use and engagement with personal devices; they do not need to change their behavior or actively engage with the study. During the active phase, we also did ecological momentary assessment (EMA) [37] 5 times a day. In the second phase of the study, the passive phase, which lasted for up to 1 year, passive data were collected from smartphones (Textbox 1). However, actigraph, bed sensor, and EMA data were no longer collected. Participants received a prompt every 2 weeks on their smartphones to fill out a PHQ-9. They were also presented with PHQ-9 at baseline. The division of the study into active and passive phases was implemented to minimize the risk of study fatigue and burden on participants.

**Raw features description.**

**Sensors and raw features**

- Accelerometer: x-axis, y-axis, z-axis, and accuracy
- App: foreground use, notifications, and crashes
- Battery: battery level, battery status, and battery health
- Call: type (incoming, outgoing, and missed) and duration
- SMS text messages: type (incoming and outgoing)
- Location: latitude, longitude, speed, and accuracy
- Screen: status (on, off, lock, and unlock)
- Actigraph: activity count and status (active, rest, and sleep)
- Murata bed sensor: heart rate, heart rate variability, respiration rate, and stroke volume

**Figure 1:**
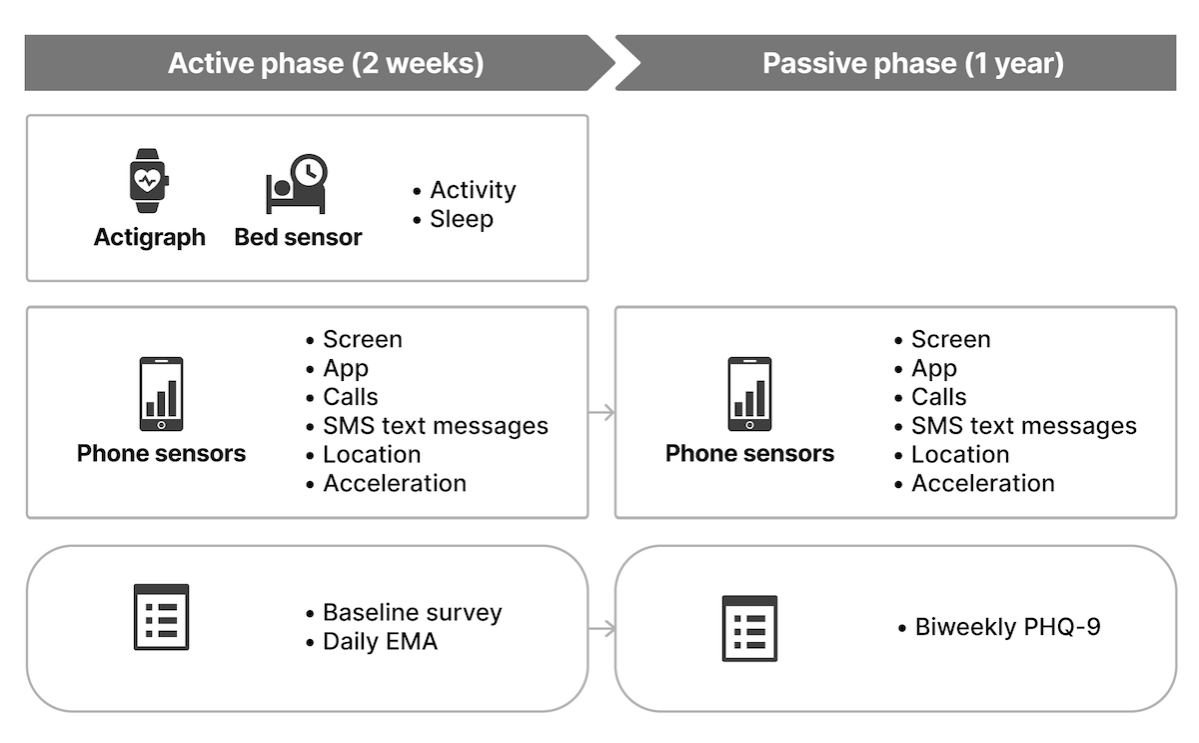
Data collection procedure of the Mobile Monitoring of Mood study. EMA: ecologic momentary assessment; PHQ-9: 9-item Patient Health Questionnaire.

The actigraphs we used did not need to be recharged for 14 days, allowing us to provide fully charged devices to participants without recharging during the active phase. In addition, PHQ-9 asks about depression symptoms in the past 2 weeks, which made the 2-week period a natural choice for the length of our active phase. Given the time frames of when people typically change their smartphones or the operating system on the smartphones, we chose the length of 1 year for the passive phase of the study. The participants could withdraw from the study at any time. Figure 1 demonstrates the study’s timeline.

### Participants and Recruitment

#### MoMo-Mood Pilot

A total of 23 healthy controls and 14 patients with MDD were recruited. One control was excluded from the study for having a baseline PHQ-9 score that fell in the subclinical depression range. The healthy control group included 17 female participants and 5 male participants, with an average age of 30.5 (SD 10.4) years. The patient group consisted of 8 female participants and 5 male participants. The average age for the patients was 32.4 (SD 12.2) years. One patient had not entered their age and sex information in the forms. In the pilot study, for practical reasons, we asked patients to stay in the study for 6 months, and we asked the healthy controls to stay in the study for 1 year.

#### MoMo-Mood

In this study, a total of 151 participants were recruited from four different groups: (1) healthy controls (n=30), (2) patients with MDD (n=76), (3) patients with BPD (n=24), and (4) patients with BD (n=21). The participants were invited to stay in the study for a maximum of 1 year. The length of involvement differed; therefore, data provided by each participant varied widely, from days to the entire year. Participants were enrolled continuously, entering and leaving at different times. This work refers to the MoMo-Mood study as the *main study*.

Participants received 4 movie tickets for participation in the study, 2 at the time of enrollment and 2 after completing the active phase. They did not receive any further compensation or feedback.

### Data Collection Platform

The Non-Intrusive Individual Monitoring Architecture is a data collection platform designed for MoMo-Mood Pilot and other digital phenotyping studies [38]. This platform allows for the design of studies with various parameters and links data from different sensors. It also provides basic cleaning and preprocessing to standardize data into tabular formats for easy handling and analysis. The Non-Intrusive Individual Monitoring Architecture platform was used to conduct both studies.

### Data Sources

#### Smartphone

We used the AWARE phone app [39] to collect data from the smartphone sensors. Our team modified and adapted this open-source framework to cater to the needs of our studies. Data collected from smartphones included communication (call and text) time stamps, an anonymous identifier for contacts, time stamps of smartphone screen events (on, off, lock, and unlock), GPS data, app use, and battery status. Each record included the user’s ID, the device’s ID, and a time stamp. The smartphone also served to ask participants several questions multiple times a day during the active phase of the study and to present them with the PHQ-9 every 2 weeks during the passive phase. Textbox 1 summarizes all the raw features gathered from each sensor.

#### EMA Technique

In clinical studies, EMA measures real-time emotional states [37]. In this method, participants respond to questions about their current state on their smartphones multiple times daily. In our studies, a prompt appeared on each participant’s smartphone once in the morning, once in the evening, and 3 times at random intervals during the afternoon, within specific time ranges. These questionnaires asked participants about their psychological and physical states, physical activity, behavior, and sleep. This paper focuses on the behavioral patterns measured objectively through passive data. Results from the EMA data are reported elsewhere [40].

#### Actigraph

Actigraphs are wrist-worn devices commonly used in clinical studies (including psychiatric studies) to measure activity levels and sleep. We used Philips Actiwatch 2 devices. The data were aggregated at 30-second intervals.

#### Bed Sensor

We used ballistocardiography-based Murata SCA11H nodes (bed sensors) to measure bed acceleration. Participants were provided with these sensors and a preconfigured Wi-Fi router, enabling data transmission directly to the study server. The sensors locally analyzed data, providing heart rate, heart rate variability, respiration rate, stroke volume, and signal strength at 1 Hz frequency.

### Data Preprocessing

#### Communication and Smartphone Screen Use

The number of incoming or outgoing calls, SMS text messages, and screen use episodes were extracted hourly. The mobile app did not transfer any message content or contact identities from the device. To ensure equal evaluation of event volume and temporal patterns, we retained participants with at least 8 weeks of communication and smartphone use data for further analyses. Records from the first and last day were excluded to avoid incomplete data capture. No additional outlier filtering was performed. To analyze the weekly temporal patterns or “rhythm” of events, we aggregated these events into 1-hour bins across 8 weeks, resulting in 168 bins for weekly frequency. One event is 1 instance of a particular data type, such as 1 call or smartphone screen-on data point. The aggregated values were normalized to sum up to 1, providing a proportionate measure of event distribution over time.

#### Apps

The frequency and duration of smartphone apps use were collected based on time stamps when apps were actively used in the foreground. Each app was manually categorized to reflect its function, including “sports,” “communication,” “leisure,” “social media,” “work,” “news,” “shop,” “transport,” and “utility.” The categorization of smartphone apps was performed after data collection was completed. App use durations >10 hours (132 instances) were removed as they might represent an error in the system rather than actual app use. Similar to communication and screen use features, we limited the data time frame to 8 weeks in the analyses that used these data. The weekly rhythm of app use was then computed using the same approach applied to communication and screen time.

#### Accelerometer

Accelerometer data were recorded every 30 seconds. The magnitude was computed as the Euclidean norm of the acceleration vectors (x-axis, y-axis, and z-axis) and normalized to center around the 0 value. We used the SD of accelerometer data at hourly intervals as a proxy for physical activity, similar to prior work [41,42]. In addition, physical activity was estimated using actigraph data described in the Sleep and Activity Detection section.

#### Sleep and Activity Detection

Sleep variables were derived from actigraph and bed sensor data. For actigraph, sleep-wake epochs were scored using the algorithm proposed by Oakley [43]. Each epoch was assigned a status indicating whether the user was active, at rest, or asleep. The active hours for each day were calculated based on the total number of epochs labeled as “active.” Bedtime was determined as the first occurrence of a continuous 3-minute sleep period.

Similarly, wake time was determined as the first instance of the first 3-minute span of activity observed in a day. For bed sensors, each 1-second epoch was given a status indicating whether the bed was occupied or free. The sleep period was defined as the longest duration during which the bed was occupied. For both sensors, records on the first and last nights were excluded due to a high likelihood of being incomplete. Nights with <3 or >13 hours of sleep were omitted, similar to previous work [44]. The preprocessing step resulted in 862 actigraph days and 841 bed sensor nights.

#### Location and Mobility

Several mobility features were extracted from the GPS sensor using the Niimpy behavior data analysis toolbox [45] which we have created. The choice of features was motivated by previous studies [23,24]. Preprocessing details and feature descriptions are provided in Table S1 in Multimedia Appendix 1 [46-48].

### Statistical Analyses

Descriptive statistics were used to summarize the demographic characteristics of participants. Survival analysis was performed using the Kaplan-Meier curve [49] to assess the likelihood of staying in the study over time for different groups. The log rank test was used to compare the differences in adherence between groups. This nonparametric test is commonly used in survival analysis in the presence of censored data.

The comparative analysis of the quantity of communication and smartphone use among different groups was performed using the Mann-Whitney *U* (MWU) test. Results with a *P* value <.05 were considered significant.

Linear mixed models were used to examine the association between passive sensor data and depression severity, as measured by the PHQ-9 score. The model incorporated behavioral features derived from sensor measures as predictors of PHQ-9 scores. Mean values of sensor data were calculated over the 14-day window preceding each PHQ-9 measurement. Continuous predictors were within-person centered to ensure comparability.

### Temporal Analysis

To determine how an individual distributes a specific activity temporally, we built *weekly activity rhythms.* The rhythm was computed as the normalized distribution of the aggregated volume of variables for 8 weeks. Intradifference of rhythm was defined as the cosine distance between the average rhythm of individuals belonging to the same cohort:



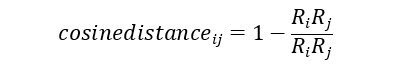



*R_i_*⋅*R_j_* represents the dot product of the activity distributions of individuals *i* and *j*, and *||R_i_||* and *||R_j_||* are these distributions’ Euclidean norms (or magnitudes).

Intuitively, individuals with similar rhythms will share a smaller distance. The MWU test was conducted to compare mean differences in intragroup distances, assessing the diversity of rhythms between groups. Post hoc correction using the Benjamini-Hochberg procedure [50] was used to control for the false detection rate during multiple tests.

### Ethical Considerations

The research protocol for both MoMo-Mood Pilot and MoMo-Mood was approved by the Helsinki and Uusimaa Hospital District’s Ethics Committee and was granted research permits by Helsinki and Uusimaa Hospital District Psychiatry (approval number § 125/2018). The approvals covered data streams, data collection platform security, and participant consent information. All data in transit were encrypted, and participant privacy was a key design value. Local IT support and the research ethics committee approved the written data security statement. Participants received detailed information about the study and the data to be collected before enrollment. All participants provided written consent. Participants were informed that they could withdraw from the study at any time. The only compensation they received was 4 movie tickets each.

## Results

### Overview

The main goal of the MoMo-Mood Pilot study was to prove the feasibility of our study design. This study had no significant issues, which led us to continue to the main study with minimal changes overall. The results presented here focus on the main study. We present the information about the participants and the population-level behavioral features from the pilot study in Table S2 in Multimedia Appendix 1.

### Participants

In the main study, a total of 164 participants were recruited. Due to technical challenges or other issues, of the 164 participants, 13 (7.9%) provided no passive data and were excluded from all analyses. Further analyses included 30 control participants and 121 participants diagnosed with major depressive episode. Among 121 patients, 76 (62.8%) were diagnosed with MDD, 21 (17.4%) with BD, and 24 (19.8%) with BPD. The pilot study included 23 controls and 14 MDD patients. All participants diagnosed with a major depressive episode were collectively categorized into a single “patient” group for clarity in the following analyses unless otherwise noted.

**Figure 2:**
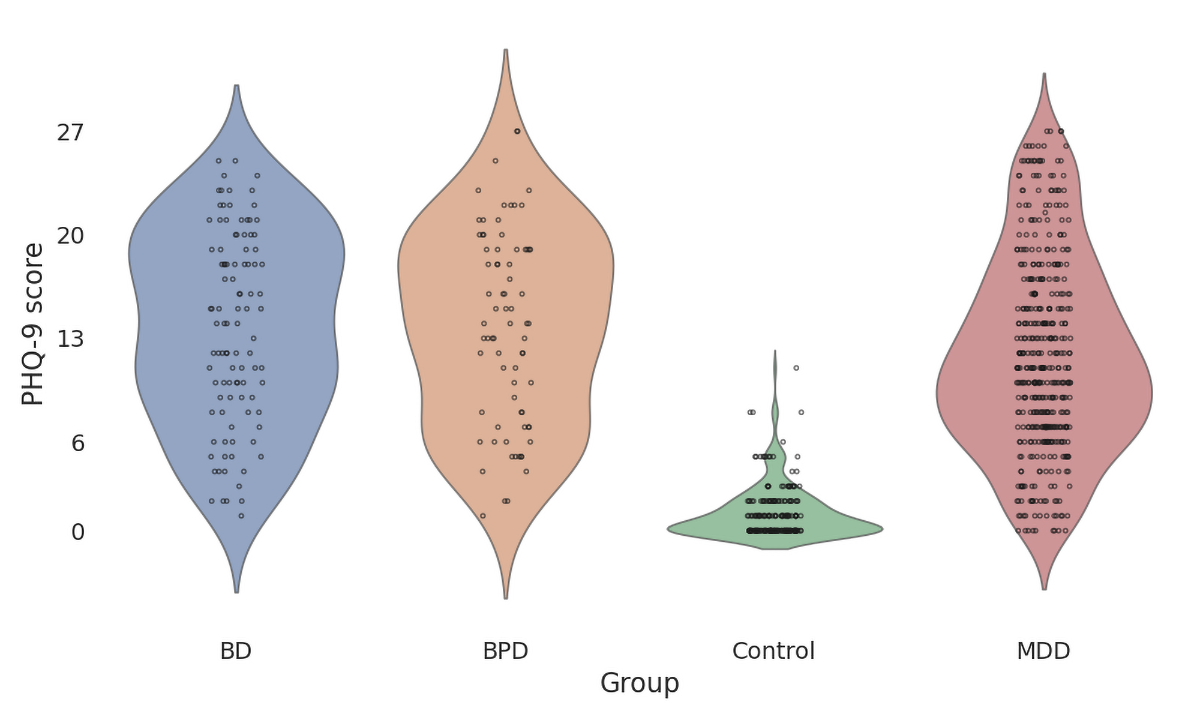
Main study 9-item Patient Health Questionnaire-9 distributions show clear differences between the control and patient groups, while differences between patient subcohorts are less prominent. BD: bipolar disorder; BPD: borderline personality disorder; MDD: major depressive disorder.

The mean age of the control group was 42 (SD 14.07) years, and the mean age of the patient group was 34.7 (SD 12.71) years. A total of 77% (23/30) of the control group were female, compared to 71.1% (86/121) in the patient group. Regarding employment, 83% (25/30) of the control group were employed full time, compared to only 9.9% (12/121) of the patient group. At baseline, the mean PHQ-9 score of the control group was 1.7 (SD 1.49). For the patient subcohorts, the baseline PHQ-9 scores were 14.6 (SD 5.41) for patients with MDD, 14.57 (SD 5.62) for patients with BPD, and 13.53 (SD 4.73) and 13.53 (SD 4.73) for patients with BD. Figure 2 shows PHQ-9 distribution for the control and patient groups. More detailed information about group-level PHQ-9 score distributions and trajectories for the active phase are reported elsewhere [14].

### RQ1: Participation Adherence

**Figure 3:**
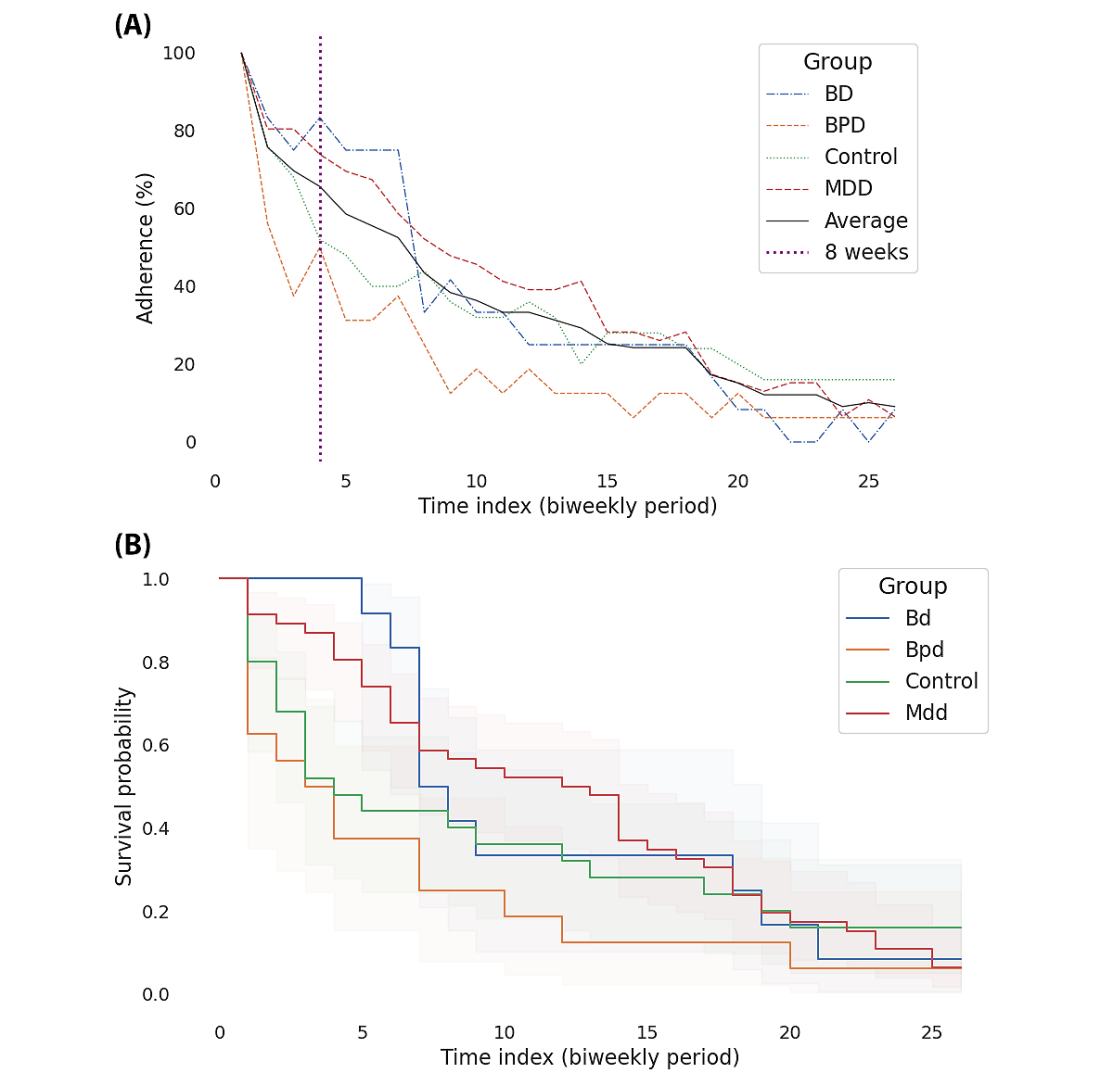
Study participants’ group average adherence proportion across the study’s passive phase. (A) Adherence was assessed by answers to the biweekly (14 days) 9-item Patient Health Questionnaire. After 8 weeks, approximately 65.7% of participants were still active in the study. The subcohort adherence rates were 50% for borderline personality disorder (BPD), 52% for controls, 73.9% for major depressive disorder (MDD), and 83.3% for bipolar disorder (BD). The adherence rates show some fluctuation due to the intermittent missing responses. (B) Kaplan-Meier survival curves depicting the probability of continued participation (survival) among different groups in the study. A participant is considered adherent if they provide biweekly period (14 days) 9-item Patient Health Questionnaire answers, starting from the beginning of the study. Notably, the curves indicate no statistically significant difference in adherence between the groups.

For this work, we defined participation adherence as the availability of the PHQ-9 responses. Adherence declined over time throughout the study; by the end of study week 8, in total 65.7% (99/151) of enrolled participants were still providing questionnaire answers and were thus considered active in the study. At this point in time, the adherence rates for different subcohorts were as follows: 50% (12/24) for patients with BPD, 52% (16/30) for controls, 74% (56/76) for patients with MDD, and 83% (17/21) for patients with BD. Figure 3A shows the adherence rates and proportion of PHQ-9 answered across the groups throughout the study’s passive phase. In addition, survival analysis using the log rank test did not reveal significant differences in adherence levels between all groups. Figure 3B shows the survival curve depicting the adherence rate assessed with PHQ-9 data. Supplementary survival analysis using smartphone battery data is presented in Figure S1 in Multimedia Appendix 1.

As an additional data quality assessment, we evaluated the data completeness by counting the proportion of missing intermittent daily passive data. We used battery data availability as a proxy for passively sensed data, considering the day as missing if no battery data were available. Combining the active and passive phases, the average passive data missingness across all participants is 10.5%, and more specifically, 4.5% for BD, 20.4% for BPD, 1.2% for control, and 14.1% for MDD groups. For each participant we calculated the total number of days with no battery data divided by the number days that participant was in the study. We then calculated the average of these percentages for all participants in a given group to derive the average percentages of passive data missingness.

### RQ2: Quantity and Temporal Patterns of Smartphone Use

To assess the difference between patients and controls in smartphone use, we measured the quantity of smartphone interaction for each group by analyzing the daily average duration of smartphone screen use and time spent on the top 5 most-used app categories. To quantify the temporal patterns of smartphone use, we used the normalized amount of interaction with the screen and these top 5 categories of apps. For both analyses, we restricted the data to an 8-week period to ensure a comparable data volume across participants and to minimize potential bias from participant dropout.

**Figure 4:**
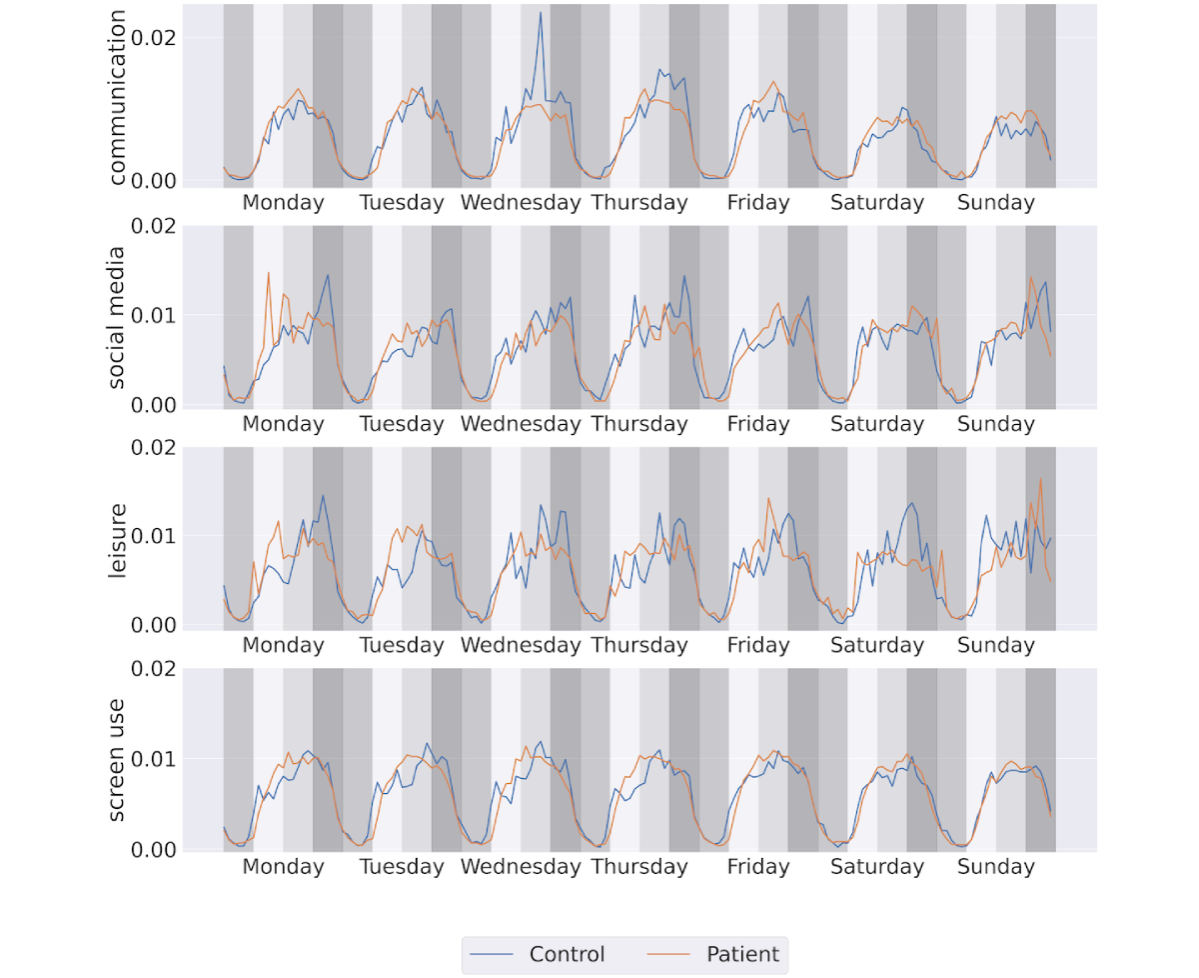
Weekly pattern of app interaction and smartphone use. The y-axis represents the normalized count of events within each 4-hour time bin over an 8-week period. Each color region denotes a 4-hour daily bin representing night (midnight to 6 AM), morning (6 AM to noon), afternoon (noon to 6 PM), and evening (6 PM to midnight).

The daily average use of the top 5 app categories and overall screen time are presented in Table 1. No significant differences were observed in the amount of time spent on each category or the overall volume of smartphone use between the groups. However, noticeable differences emerged when examining the temporal patterns of app use. As illustrated in Figure 4, controls tend to use leisure apps more frequently during the weekend, with a peak on Saturday. This pattern suggests that controls prefer to engage with entertainment apps during their free days.

**Table 1:** Daily average duration of top 5 app use categories and total time spent using the smartphone^a^.

Duration of use (min)	Control, mean (SD)	Patient, mean (SD)	*P* value
Communication	18.46 (11.65)	29.06 (36.18)	.87
Social media	31.62 (22.59)	39.41 (41.23)	.90
Leisure	18.62 (18.30)	29.20 (50.92)	.87
Games	11.50 (15.73)	13.81 (25.26)	.87
News	16.12 (42.73)	7.36 (15.33)	.90
Screen use	225.91 (113.68)	178.18 (130.92)	.55

^a^*P* values from the Mann-Whitney *U* test, adjusted to correct for multiple testing.

### RQ3: Between-Group Difference in the Quantity and Temporal Patterns of Behaviors

We used the same strategy to quantify daily communication and location patterns from smartphone sensors, sleep patterns from bed sensors, activity patterns from actigraphs, and weekly temporal communication patterns.

#### Communication

We used call and SMS text message data collected by smartphones as a proxy of social activity. We collected 46,788 incoming and outgoing calls, of which 39,491 (84.4%) were longer than 0 seconds, with an average daily incoming call duration of 6.9 (SD 17.2) minutes and average daily outgoing call duration of 7.4 (SD 19.3) minutes. In addition, we collected 46,031 SMS text messages within the same period. Again, we restricted the data to an 8-week period for this analysis to ensure comparable temporal patterns. Table 2 presents the mean (SD) of the 2 groups’ daily calls and SMS text messages. A comparison of the daily average communication between groups did not show significant differences.

**Figure 5:**
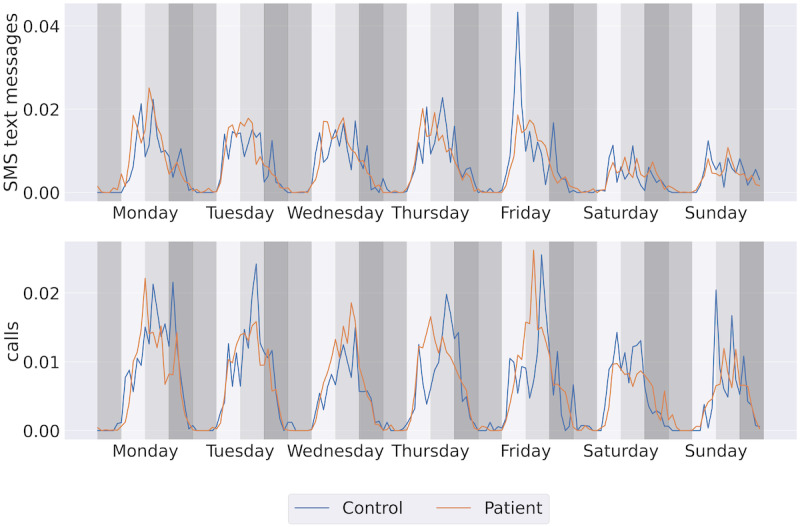
Weekly pattern of communication. The y-axis represents the normalized count of events within each 4-hour time bin over an 8-week period. Each color region denotes a 4-hour daily bin representing night (midnight to 6 AM), morning (6 AM to noon), afternoon (noon to 6 PM), and evening (6 PM to midnight).

Figure 5 displays the weekly communication rhythms for both groups. A visual inspection reveals that both groups tended to have a decreased communication volume during the weekend. Within the control group, call events were more frequently observed in the afternoon bins (noon to 6 PM).

**Table 2:** Daily average calls and SMS text messages per group for each study^a^.

	Control, mean (SD)	Patient, mean (SD)	*P* value
Number of incoming calls	0.67 (0.58)	1.26 (1.17)	.64
Number of outgoing calls	1.26 (1.21)	1.59 (1.37)	.90
Incoming call duration (min)	4.87 (6.08)	7.89 (7.36)	.64
Outgoing call duration (min)	5.55 (5.48)	7.67 (9.94)	.94
Number of incoming SMS text messages	1.24 (0.79)	1.48 (1.23)	.94
Number of outgoing SMS text messages	0.66 (0.98)	0.95 (1.27)	.69

^a^*P* values from the Mann-Whitney *U* test were adjusted for multiple tests.

#### Sleep and Physical Activity

**Figure 6:**
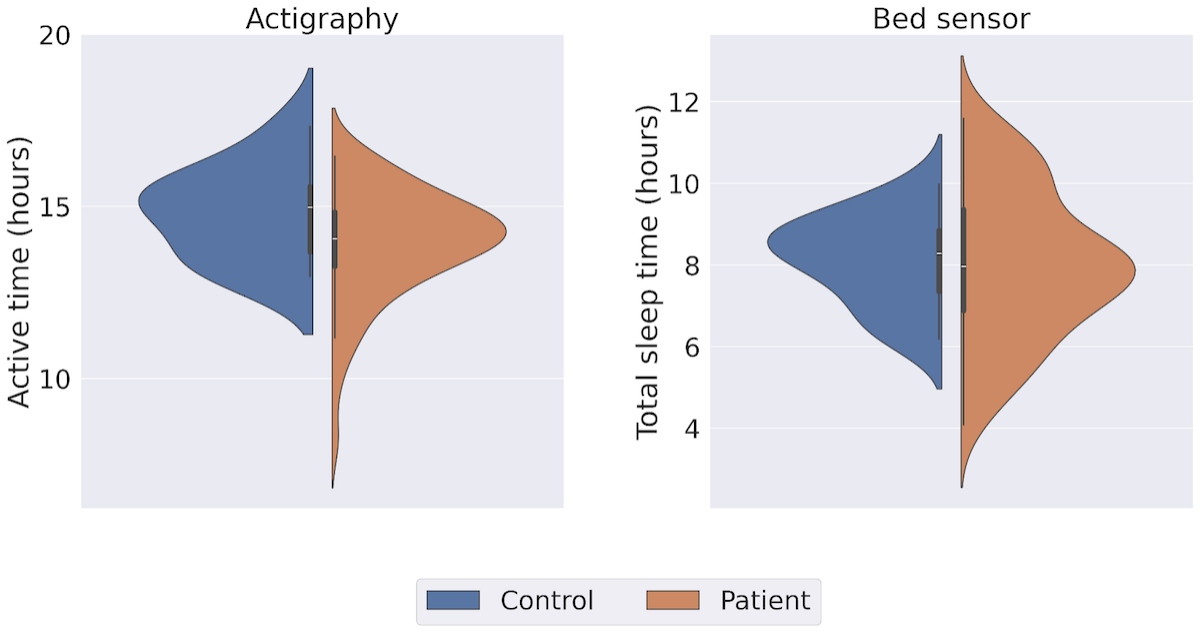
Sleep variables extracted from actigraph and bed sensor.

We examined all participants’ sleep and physical activity patterns via features extracted from the actigraph and the bed sensor. We look at these 2 behaviors together as they complement each other throughout the day. We measured the sleep duration from the bed sensor and the active hours from the actigraphy. The daily average active hours and sleep duration of each group in each study are presented in Figure 6. We found no between-group difference in sleep duration (patient: mean 7.99, SD 1.80; control: mean 8.13, SD 1.05; MWU test *P*=.57). Similarly, active hours showed no significant difference between groups (patient: mean 13.93, SD 1.57; control: mean 14.80, SD 1.30; MWU test *P*=.14).

**Figure 7:**
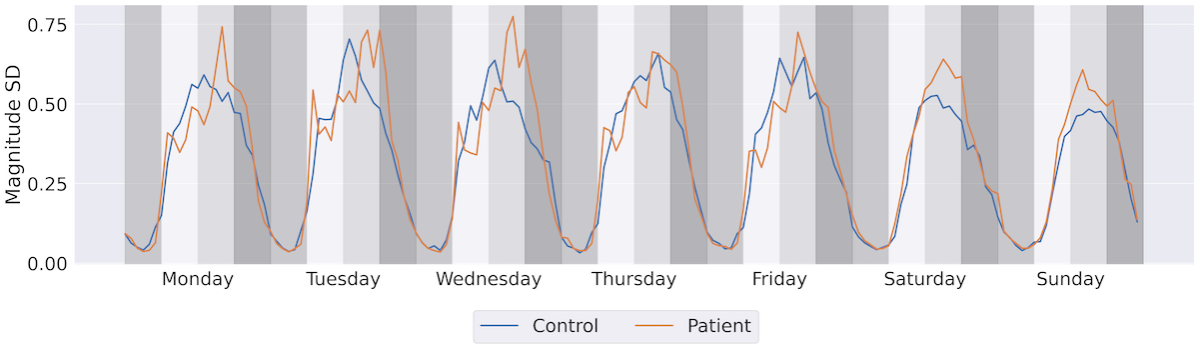
Weekly pattern of physical activity intensity measured by the SD of the accelerometer’s magnitude.

We measured the intensity of physical activity using the SD of the phone’s magnitude computed from the accelerometer sensor. Again, we found no significant difference in daily physical activity intensity between the groups (patient: mean 0.60, SD 0.32; control: mean 0.58, SD 0.34, MWU test *P*=.35). The temporal trend (Figure 7) showed a general decrease in activity intensity toward the weekend. This reduction is more noticeable in the control group.

#### Mobility and Location

**Figure 8:**
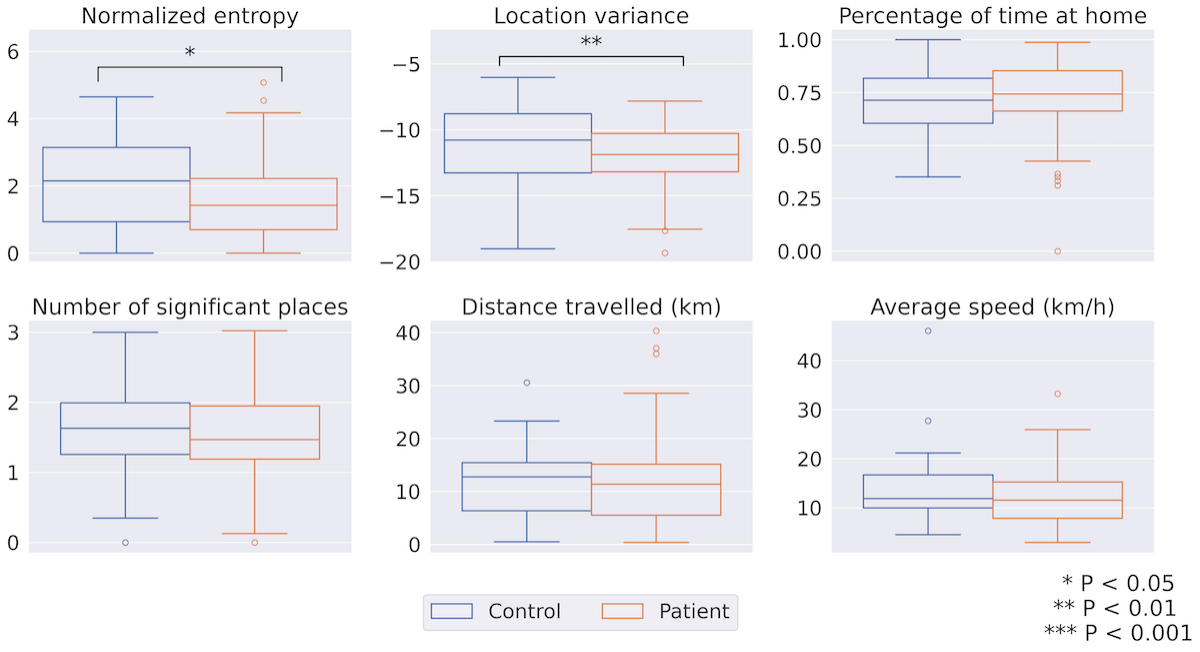
Weekday location features. Asterisks denote significance.

Due to the inherent difference in mobility patterns during workdays and weekends, we separated the features for these 2 time frames. Figure 8 demonstrates the weekday location features of the 2 groups. Significant differences were observed in weekday location features. Specifically, both location variance (patient: mean –11.91, SD 2.50; control: mean –10.04, SD 2.73; MWU test *P*=.004) and normalized entropy (patient: mean 1.57, SD 1.10; control: mean 2.10, SD 1.38; MWU test *P*=.05) were lower in patients. However, no significant differences in weekend location features were detected. When the analysis was restricted to participants with work duties, only the weekday location variance remained significant (patient: mean –11.14, SD 2.08; control: mean –10.00, SD 2.80; MWU test *P*=.05). Weekend-weekday location features’ values are presented Table S3 in Multimedia Appendix 1.

### RQ4: Similarity in Temporal Rhythms of Smartphone Use, Social Activity, and Physical Activity Within Subcohorts

We examined the extent to which participants within the same group exhibited similar temporal rhythms of smartphone use patterns. Higher homogeneity of behavior within a group indicates that members exhibit similar behaviors. To compare within-group similarity, we calculated the cosine distance between individuals’ average weekly rhythms within each group (patients-patients and controls-controls). We applied the MWU test to determine significant differences between groups’ mean distances, thus evaluating internal group consistency. The analysis timeframe was limited to 8 weeks to ensure a consistent evaluation of temporal patterns.

**Figure 9:**
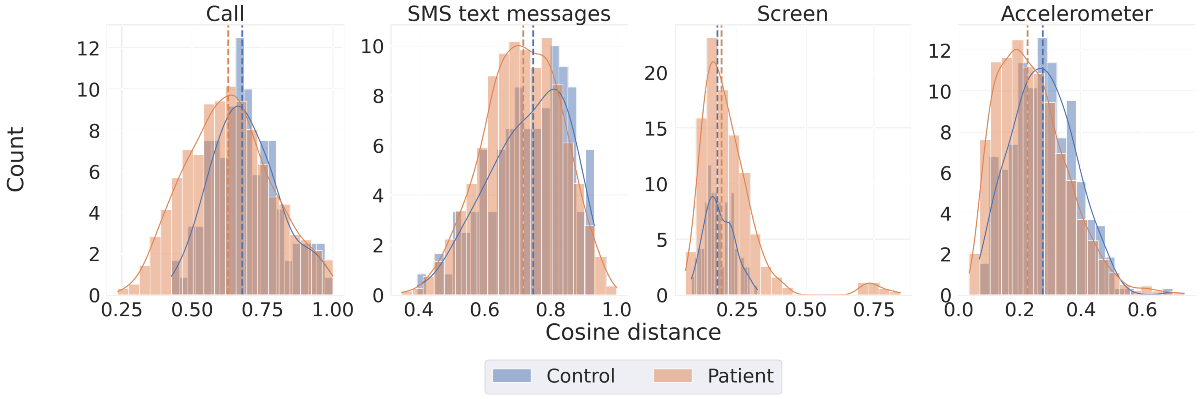
Histogram representing the intradifference of weekly rhythm measured by cosine distance. Smaller distances indicate more intraindividual similarity.

Figure 9 shows the relative within-group distance for each of the groups. Patients established a more homogenous communication rhythm than the controls, meaning that their time allocation for communication was more similar to one another compared to how the healthy controls compared to each other (MWU test, call: *P*<.001; SMS text message: *P*=.04). Because most patients did not have full-time jobs at the time of the study, we also examined the potential confounders by composing the same analysis only on the participants who had full-time jobs. Nonetheless, the previous results persisted, meaning employment did not play a significant role in maintaining communication rhythms.

By contrast, patients exhibited less consistent smartphone use rhythms compared to controls (MWU test *P*=.002). However, among participants with full-time jobs, controls displayed less consistency in smartphone use than patients (MWU test *P*=.002). Patients exhibited more consistency in activity rhythms captured by accelerometers than controls, across both the entire sample and the group of full-time employed individuals (MWU test *P*<.001).

### RQ5: Sensor Measures as Predictors of Depression Severity

In addition to analyzing the differences in sensor measures between participant groups, we also examined the association between these measures and the severity of depressive symptoms. To this end, we fitted a linear mixed model with each sensor-derived feature as a predictor, the raw PHQ-9 score as the response variable, and participant ID as the grouping factor. Model estimates are presented in Table 3.

**Table 3:** Behavioral factors predicting the severity of depression symptoms^a^.

		Estimates (95% CI)		*P* value
**Model 1: communication**				
	Incoming call duration—W	–0.08 (–0.12 to –0.04)	*<.001*	
	Outgoing call duration—W	0.05 (0.00-0.09)	*.02*	
	Number of incoming SMS text messages	0.31 (–0.11 to 0.77)	.14	
	Number of outgoing SMS text messages	–0.13 (–0.59 to 0.31)	.56	
**Model 2: location**				
	Location log variance—W	–0.10 (–0.44 to 0.18)	.48	
	Normalized entropy—W	0.55 (–0.03 to 1.11)	.08	
	Distance traveled—W	–0.00 (–0.00 to 0.00)	.12	
	SPs	–0.11 (–1.33 to 1.19)	.87	
	Percentage of time at home	0.67 (–2.71 to 4.06)	.68	
**Model 3: duration of phone and app use**				
	Social media—W	–0.01 (–0.02 to 0.00)	.24	
	Communication—W	0.00 (–0.01 to 0.02)	.88	
	Games—W	–0.01 (–0.03 to 0.01)	.34	
	Leisure—W	–0.01 (–0.02 to 0.00)	.14	
	Screen use time—W	0.01 (–0.00 to 0.01)	.10	
**Model 4: accelerometer**				
	Magnitude SD morning	–2.05 (–4.18 to –0.20)	*.02*	
	Magnitude SD afternoon	0.30 (–1.49 to 2.15)	.82	
	Magnitude SD evening	0.29 (–1.94 to 2.32)	.78	
	Magnitude SD night	–0.94 (–6.77 to 3.65)	.66	

^a^Within-person mean-centered predictors are suffixed with *W*. The unit for duration variables is minutes.

Our analysis of sensor data revealed notable associations in communication and physical activity patterns related to depression symptoms. For communication patterns, the duration of incoming calls was negatively associated with depression symptoms (β=–0.08, 95% CI –0.12 to –0.04; *P*<.001), indicating that shorter incoming call durations were linked to higher depression severity. In contrast, the duration of outgoing calls was positively associated with depression symptoms (β=0.05, 95% CI 0-0.09; *P*=.02), suggesting that longer outgoing call durations were associated with greater depression severity. For physical activity data, the SD of activity magnitude in the morning was negatively associated with depression symptoms (β=–2.05, 95% CI –4.18 to –0.20; *P*=.02).

## Discussion

### Principal Findings

In the MoMo-Mood study, there were no significant differences in adherence between patients and healthy controls. However, the overall adherence rates for all groups were low. Our analysis of smartphone use patterns did not reveal significant differences between the groups in terms of the daily quantity of smartphone use. However, the weekly temporal patterns revealed distinct preferences, such that the control group typically made or received calls in the afternoon. Furthermore, we initially hypothesized that patients would exhibit notable differences in activities and behaviors crucial for assessing mood disorder symptoms or associated psychosocial disability across 4 domains: social activity, sleep, physical activity, and mobility. Our findings partially supported this hypothesis, as we found significant differences in mobility and location patterns, particularly in terms of normalized entropy and variance of locations during weekdays. However, the groups showed no notable differences in sleep, social, and physical activity patterns.

The within-group temporal rhythm analysis revealed more nuanced findings. The patient group demonstrated more similar communication and physical activity rhythms to one another than the control group. By contrast, patients showed more varied rhythms in smartphone screen use. Interestingly, this trend for the smartphone screen patterns reversed when we took employment status into account.

Using passive data to predict the severity of depression symptoms, we found that depression severity was negatively associated with the duration of incoming calls and accelerometer magnitude variability but positively associated with the duration of outgoing calls.

### Implications

The similarity in smartphone engagement between patients and controls validates the potential of smartphone data as a reliable source for finding new behavioral markers. Notably, despite being mildly to moderately clinically depressed, the patients remained actively engaged with their smartphones. Furthermore, the analysis supports the value of smartphone data in capturing temporal behavioral patterns and reveals differences in behavioral rhythms, including communication and screen use patterns. However, low adherence rates limit the generalizability of this methodology.

In contrast to prior research [10], our study did not confirm the significance of sleep duration in detecting depression symptoms, although it is well known that both insomnia and hypersomnia are symptoms of depression [21]. In the future, we will conduct a more fine-grained analysis focusing on the disturbances and individual differences in sleep.

Our exploration of weekday and weekend mobility patterns and location features revealed a significant difference in weekday normalized entropy and location variance between the groups. These findings are consistent with prior research, such as the studies by Saeb et al [23,24], which identified a significant correlation between depression scores and location features, including location variance and location entropy. The importance of mobility and location data is also supported by previous literature [10,51,52], recognizing these data as crucial components in modeling depression severity.

When examining the similarity of behavior among patients and among healthy controls, we found that, across 3 different activity types—communication via calls and SMS text messages, and smartphone acceleration—patients displayed more similar behaviors to one another. These results persisted even when controlling for the participant’s employment status. This highlights the importance of temporality and temporal behavioral features as behavioral markers for mood disorders. The tendency for patients to engage in communication efforts or physical activities at similar times of the day or week may suggest common behavioral trends among patients. For example, this could manifest as an increased number of calls in the morning or a noticeable reduction in physical activity during that time compared to the control group. Such patterns might indicate shared disruptions in circadian rhythms, where patients struggle with energy and motivation early in the day. Alternatively, it may reflect a more structured or predictable routine as a coping mechanism, helping patients manage their symptoms.

It is essential to keep in mind that digital phenotyping studies aim to capture subtle differences and patterns in the behavior of patients and healthy controls, which can possibly be missed even by trained clinicians. Any major behavioral differences between these groups have naturally already been noticed and studied by experts in psychiatry. Our study adds to the body of knowledge in digital phenotyping. It points out essential yet subtle features that need further research and can potentially be used to expand the behavioral symptomology of mood disorders.

Our findings provide valuable longitudinal insights and generalizability by representing different types of patients with major depression. It is important to note that most of our patients experienced from mild to moderate clinical depression, representing the range of severity of most outpatients with depression in health care. However, the inclusion of psychiatric inpatients with severe or psychotic depression, or individuals with BD experiencing manic episodes, might have revealed more marked deviations from standard behavioral patterns. Furthermore, patients were receiving treatment, and it is likely that, for example, the observed sleep symptoms were influenced by ongoing pharmacotherapy.

Our results can help future studies improve their designs, increase adherence, and focus on the most promising digital traces and meaningful features. Ultimately, these findings, combined with insights from future similar studies, may lead to the development of tools for monitoring and in-time intervention for patients with mood disorders.

### Limitations

The research is limited by incomplete data, declining participant adherence, and increasing dropout rates as the study progresses. These are common challenges in digital phenotyping studies that may decrease the study’s analytical power, introduce bias, and obscure longitudinal patterns and interactions in the data. The sources of poor adherence and incomplete data collection may stem from technical issues, participant burden, lack of motivation, or health status. Other digital phenotyping studies have similarly reported adherence of various degrees, ranging from 65.3% smartphone data completeness (a study with 334 patients with MDD monitored for 12 weeks [53]) to 99% completeness (a study with 29 patients diagnosed with BP monitored for 1 year [54]). The adherence may also vary between the study cohorts. For example, a study [52] reported average adherence levels of 84.8% for patients with BD and 66.8% for the healthy controls for daily smartphone-reported self-assessments collected over 9 months. However, in our study, adherence remained similar for different subcohorts. Our participants were not followed up on or engaged with after they completed the active phase of the study (approximately 2 weeks after the enrollment). The lack of further follow-up or feedback to the participants has likely played an essential role in our dropout rates.

Our study does not include patients with severe depression, inpatients, and patients with psychotic depression. This limits the applicability of our findings for these cases.

This study is limited by the data types that we could collect. Thus, it does not capture the full extent of a person’s communication (eg, communication via social media or messaging apps). Furthermore, for devices such as bed sensors and actigraphs, we relied on the preprocessed data provided by the manufacturer, which can potentially introduce unknown errors and biases.

### Future Research

We have collected a very rich dataset that requires much more extensive exploration. This paper provides an overview of the study and compares behavioral patterns at the group level for the passively collected data from smartphones, bed sensors, and actigraphs. Future research should include more detailed analysis at the individual level, a deeper exploration of the parameters highlighted in this study, and an examination of the relationship between passive data and subjective data collected through validated clinical questionnaires.

The results of this study show that the mobile behavioral data, including features from smartphone use, communication, location, mobility, and sleep, collected within this study can be further used to predict and monitor the patients’ depression. The group-level differences observed in this study, based on the comparison of rhythms, suggest the importance of focusing on extracting temporal features from behavioral data. Therefore, further studies are required to validate these findings. We have released an open-source behavioral data analysis toolbox, Niimpy [45], to facilitate the studies and encourage researchers to use and further develop it. Furthermore, we propose that future analyses use personalized statistical and machine learning models, accounting for the differences in behavior and sociodemographic information (eg, participant’ age, sex, and work status). In the future, we will extend our analyses and explore the possibility of predicting the future mood state and severity of depression from features extracted from the passively collected data.

### Conclusions

This study demonstrates the feasibility of harnessing data from a cohort of patients with different types of clinically diagnosed depressive syndromes. It also shows the important features and data streams for future analyses of behavioral markers of mood disorders. However, among outpatients with mild to moderate depressive disorders, the group-level differences from healthy controls in any of the modalities remained relatively modest, despite the demonstration of within-individual variations in behavioral patterns linked to the severity of depression. Overall, the sensors used in passive monitoring may more readily detect gross observable behavioral abnormalities, which may emerge only in the epidemiologically rarer severe range of depression or in mania. Therefore, future studies need to be able to combine data from multiple domains and modalities to detect more subtle differences, identify individualized signatures, and combine passive data with clinical ratings.

## Data Availability

Due to the highly sensitive and private nature, the data collected in these studies are not publicly archived or available. Our research permit does not allow the free availability of these data. Accessing the data could potentially be possible through collaboration requests.

## Appendix

Multimedia Appendix 1 Supplementary analyses.

## Abbreviations

BD: bipolar disorder
BPD: borderline personality disorder
EMA: ecological momentary assessment
MDD: major depressive disorder
MoMo-Mood: Mobile Monitoring of Mood
MWU: Mann-Whitney U
PHQ-9: 9-item Patient Health Questionnaire
RQ: research question
