# Supplementary material for: Multimodal Digital Phenotyping Study in Patients With Major Depressive Episodes and Healthy Controls (Mobile Monitoring of Mood): Observational Longitudinal Study
Source: JMIR Ment Health. 2025 Feb 21;12:e63622. doi: 10.2196/63622 (PMC11890149; doi:10.2196/63622)
Supplement: Multimedia Appendix 1 [file mental_v12i1e63622_app1.docx]

**Location features description**

A significant challenge in collecting GPS data is the prevalence of missing data and outlier values. Less than 1% of the data points consisted of the *(longitude, latitude) = (0, 0)* coordinate, which fell in the middle of the Atlantic Ocean and was not a plausible location for our participants. Furthermore, a proportion of data points derived from the WiFi signal contain very high-speed values (>10^2^ m/s). Therefore, we retained only data points derived from GPS sources and systematically eliminated outliers to ensure data validity. Following this process, 6,287,582 data points were filtered out from the original count of 11,346,218, resulting in a total of 5,058,636 data points being retained and eligible for further analysis. For the distance traveled feature, we filter the outliers by accepting only daily trips less than 100km.

| Feature name | Description |
| --- | --- |
| Distance traveled | Geodesic distance traveled in meters, computed as the summation of distances between all consecutive location data points. [[52]](https://www.zotero.org/google-docs/?g4hOR9) |
| Significant places | Number of location clusters found by the DBSCAN algorithm [[53]](https://www.zotero.org/google-docs/?YkDmOL) |
| Normalized entropy | Information theoretical entropy [[54]](https://www.zotero.org/google-docs/?B3ZFNf), which measures how each participant’s time was distributed over different significant places |
| Proportion of time spent at home | The fraction of time spent at home in a day. Home is considered to be the significant place a subject spends the most time between midnight and 6 AM. |
| Speed mean | The average instantaneous speed obtained at each GPS point, calculated as the geodesic distance between successive latitude and longitude coordinates over the elapsed time, is calculated as follows.  $v = \frac{geodesic((lat_{t-1}, lon_{t-1}), (lat_{t}, lon_{t}))}{\Delta t}$ |
| Location variance | Log of the sum of the variance of latitude and longitude  $variance= log(\sigma_{lat}^{2} + \sigma_{lon}^{2})$ |

*Table S1:* Location and mobility features description.

##

### **Results from the pilot study**

In the pilot study, the mean age of the control group was 30.59 ± 10.54 years, and the mean age of the patient group was 32.38 ± 12.21. 65.21% of the control group were female, compared to 57.14% in the patient group; like the main study, employment and study engagement were high among the control group, with 95.96% either employed or studying, compared to 42.86% in the patient group. At baseline, the mean PHQ-9 score of the control group was 1.64 ± 1.65, and for the patient group was 13.78 ± 6.09. Table A1 presents the population-level behavioral features for the patient and control groups in the pilot study.

|  | | | | | | | | Control (mean, sd) | | | | Patient (Mean + sd) | | | |
| --- | --- | --- | --- | --- | --- | --- | --- | --- | --- | --- | --- | --- | --- | --- | --- |
| **Communication** | | | | | | | | | | | | | | | |
| Incoming call duration (minutes) | | | | | | | | 2.93 +- 2.57 | | | | 8.18 +- 7.14 | | | |
| Outgoing call duration (minutes) | | | | | | | | 2.96 +- 2.58 | | | | 3.78 +- 4.67 | | | |
| Num. of incoming call | | | | | | | | 0.69 +- 0.40 | | | | 1.33 +- 1.75 | | | |
| Num. of outgoing call | | | | | | | | 1.19 +- 0.92 | | | | 1.48 +- 2.10 | | | |
| Num. of incoming SMS | | | | | | | | 1.20 +- 1.12 | | | | 1.11 +- 0.85 | | | |
| Num. of outgoing SMS | | | | | | | | 0.66 +- 0.84 | | | | 0.82 +- 0.82 | | | |
| **Location - Weekday** | | | | | | | | | | | | | | | |
| Location log variance | | | | | | | | -9.88 ± 2.31 | | | | -10.26 ± 2.06 | | | |
| Normalized entropy | | | | | | | | 2.47 ± 1.40 | | | | 1.64 ± 0.94 | | | |
| Distance traveled (km) | | | | | | | | 14.79 ± 8.96 | | | | 12.54 ± 8.07 | | | |
| SPs | | | | | | | | 1.80 ± 0.86 | | | | 1.64 ± 0.65 | | | |
| % time at home | | | | | | | | 0.60 ± 0.14 | | | | 0.70 ± 0.12 | | | |
| **Location - Weekend** | | | | | | | | | | | | | | | |
| Location log variance | | | | | | | | -10.55 ± 1.89 | | | | -9.30 ± 1.15 | | | |
| Normalized entropy | | | | | | | | 2.02 ± 1.19 | | | | 1.81 ± 1.67 | | | |
| Distance traveled (km) | | | | | | | | 14.02 ± 8.52 | | | | 13.54 ± 13.11 | | | |
| SPs | | | | | | | | 1.68 ± 0.76 | | | | 1.63 ± 0.87 | | | |
| % time at home | | | | | | | | 0.64 ± 0.16 | | | | 0.73 ± 0.18 | | | |
| **Phone usage** | | | | | | | | | | | | | | | |
| Screen use time (minute) | | | | | | | | 82.62 +- 40.8 | | | | 121.49 +- 87.2 | | | |
|  | | | | | | | | | | | | | | | |
| Active hours (Actigraph) | | | | | | | | 14.82 +- 1.18 | | | | 14.10 +- 1.24 | | | |
| Sleep duration (hours, Bed sensor) | | | | | | | | 7.65+- 1.10 | | | | 8.23 +- 2.18 | | | |

Table S2: MoMo-Mood Pilot study population-level behavioral features.

**Comparison of Weekday and Weekend Location Features by Groups**

|  | Weekday | | | Weekend | | |
| --- | --- | --- | --- | --- | --- | --- |
|  | Control | Patient | *P value* | Control | Patient | *P value* |
| Location variance | -10.22 ± 2.70 | -11.97 ± 2.56 | *.006*** | -13.08 ±2.83 | -12.49 ± 3.23 | .34 |
| Normalized entropy | 2.05 ± 1.39 | 1.52 ± 1.12 | *.05** | 1.07 ± 0.89 | 1.35 ± 1.14 | .34 |
| % Time at home | 0.71 ± 0.18 | 0.73 ± 0.18 | .57 | 0.83 ± 0.12 | 0.76 ± 0.18 | .15 |
| Number of SPs | 1.62 ± 0.65 | 1.49 ± 0.65 | .30 | 1.31 ± 0.42 | 1.41 ± 0.63 | .40 |
| Average speed (km/h) | 14.32 ± 8.35 | 12.48 ± 5.91 | .36 | 12.66 ± 8.58 | 10.88 ± 6.00 | .34 |
| Total distance (km) | 11.69 ± 7.29 | 11.83 ± 7.97 | .91 | 8.32± 5.99 | 11.32 ± 9.75 | .19 |

*Table S3:* Description of location features of the main study. Weekday and weekend features were calculated separately for each group. The *p*-values are from MWU tests.

## **Survival analysis based on passive data**


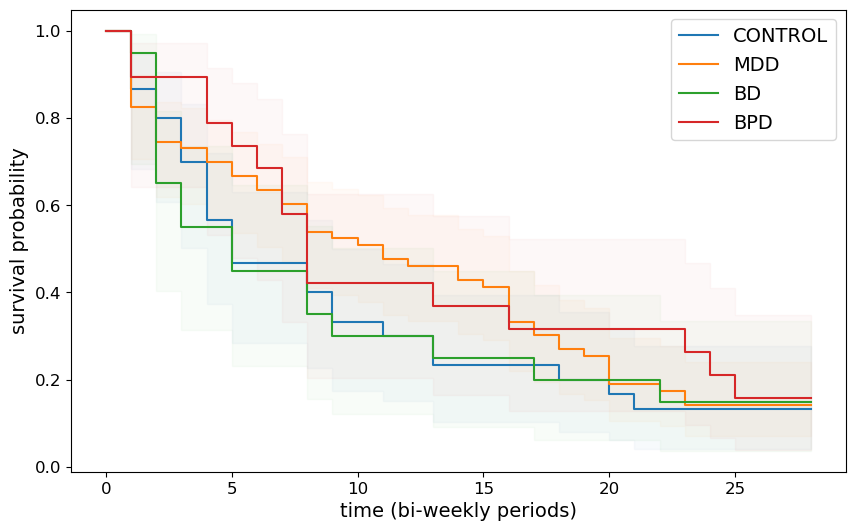


*Figure A1:* Kaplan-Meier survival curves depicting the probability of continued participation (survival) among different subcohorts in the study. To be considered adherent, a participant must provide battery data for at least ten days within each bi-weekly period (14 days), starting from the beginning of the study. Notably, the curves indicate no statistically significant difference in adherence between the groups.
